# Induced resistance to herbivory and the intelligent plant

**DOI:** 10.1080/15592324.2024.2345985

**Published:** 2024-04-30

**Authors:** André Kessler, Michael B. Mueller

**Affiliations:** Cornell University, Department of Ecology and Evolutionary Biology, Ithaca, NY, USA

**Keywords:** Plant–insect interactions, plant defense, secondary metabolite production, signaling pathway crosstalk, chemical defense, immunological memory

## Abstract

Plant induced responses to environmental stressors are increasingly studied in a behavioral ecology context. This is particularly true for plant induced responses to herbivory that mediate direct and indirect defenses, and tolerance. These seemingly adaptive alterations of plant defense phenotypes in the context of other environmental conditions have led to the discussion of such responses as intelligent behavior. Here we consider the concept of plant intelligence and some of its predictions for chemical information transfer in plant interaction with other organisms. Within this framework, the flow, perception, integration, and storage of environmental information are considered tunable dials that allow plants to respond adaptively to attacking herbivores while integrating past experiences and environmental cues that are predictive of future conditions. The predictive value of environmental information and the costs of acting on false information are important drivers of the evolution of plant responses to herbivory. We identify integrative priming of defense responses as a mechanism that allows plants to mitigate potential costs associated with acting on false information. The priming mechanisms provide short- and long-term memory that facilitates the integration of environmental cues without imposing significant costs. Finally, we discuss the ecological and evolutionary prediction of the plant intelligence hypothesis.

## Introduction

As sessile organisms rooted to a substrate plants’ fitness depends on their ability to adjust growth and metabolism to fluctuating environmental conditions. While the phenotype results from a genotype by environment interaction,^1^ the extent to which a plant can express phenotypic plasticity is also genetically determined.^2^ Thus, different biotic and abiotic environmental conditions have been found not only to result in differential growth forms resulting from altered developmental trajectories but also to include reversible metabolic changes that can have multiple ecological functions, most notably those that mediate interactions of plants with other organisms, such as pathogens, herbivores, various mutualists, and competing neighbors.^3,4^ In consequence, plant-induced responses that allow plants to plastically adjust their morphological and metabolic phenotypes to an ever-changing environment are increasingly studied in a behavioral ecology context.^5^ By integrating external and internal information to best match their phenotype with the current environment, plants can maximize their performance and fitness as sessile organisms in a highly dynamic environment.^3^ In extension, plants are now considered to exhibit a range of basic and specialized behavioral categories, such as learning, memory, and context-dependent adjustments of behavioral patterns (intelligence).^6,7^ For example, plants can experience fundamental changes to their primary and secondary metabolism in response to herbivore attacks.^8^ Many of the herbivory-induced changes to secondary metabolism, such as the increased production of repellant, toxic, antidigestive, and antinutritive compounds, can result in higher (induced) resistance to subsequent herbivory.^8^ At the same time, induced secondary metabolite production can provide information to and facilitate the prey-search behavior of natural enemies of herbivores, mediating so-called indirect defenses.^9–11^ Such behavioral adjustments of plants’ metabolic phenotypes to environmental stresses can have dramatic effects on the outcome of species interactions, and, by extension, on population, community, and ecosystem dynamics.^12–14^ This is because plants are the primary producers and alterations to their metabolism can be predicted to disproportionately affect the flow of energy and resources through the trophic cascade. But how impactful can plant induced responses, for example, to herbivory, or plant behavior really be in influencing ecological dynamics? Or, more pragmatically, is there a value in studying induced responses to environmental stresses in a behavioral context to better understand ecological and evolutionary processes?

On this very basic level, the research community studying plant–animal interactions and specifically induced responses to herbivory has long been using the behavior framework implicitly or explicitly when formulating the hypotheses associated with plant defense theory. However, the application of more specific behavioral properties to plant ecology, such as cognition and intelligence, is just beginning and is highly controversial. Here we consider the concept of intelligent behavior in plants when interacting with herbivores as a potential conceptual tool in the study of plant–herbivore interactions. However, the mere definition of intelligence provides the commonest hurdle to its study.^15^

It has long been evident that our anthropocentric worldview on intelligence, ridden by “brain chauvinism” and “neuro-centrism”, has limited the consideration of behavioral expressions, such as cognition, intention, or intelligence as properties that are potentially inherent to all life.^7^ The idea that plants behave intelligently is not new.^7,16–18^ Darwin already remarked on how his observations on the movement of plant roots resembled both the “brain” and “sense organs” of animals.^19^ However, more recently, controversy emerged about the definition of intelligence and the necessity of a centralized nervous system structure to facilitate intentional behavior or intelligence in any organism, specifically in plants.

The apparent lack of an agreeable definition for intelligence is currently one of the most significant barriers to expanding the study of intelligent behavioral expression to brainless creatures, such as plants. This is certainly different from other previously or currently debated concepts in science. For example, there is broad agreement on the concept of gravity, while the actual physical mechanism may have been subject to discussion.^20^ For intelligence, on the other hand, one can find more than 70 definitions that are tinged by experimental, technical, and philosophical biases of different research fields that aim to study it.^7,21^ Undoubtedly one such bias is the notion that intelligent behavior requires a centralized nervous system and thus needs to be based on signal transduction by action potentials aided by synaptic connections between nerve cells. The direct application of that concept to plants has led to a periodically reemerging and lively discussion, mostly proven *ad absurdum* by what we currently know about plant physiology.^22–31^ To be fair, “plant neurophysiology” has taught us much about the potential role of electric signaling in plant signal transduction cascades that link the environment to metabolic responses; however, it has also diverted the attention from the possibility that there might be forms of intelligence based on different mechanisms of processing information.

The advent of artificial intelligence brought along a growing acknowledgment that systems, even those lacking neurons and nervous systems, may exhibit signs reminiscent of intelligence. However, if we wish to discuss intelligence honestly and constructively, it requires a more general definition of what intelligence is (Box 1). In an attempt to synthesize the available definitions of intelligence from both psychologists and artificial intelligence researchers, Legg and Hunter (2007) present the “universal intelligence” definition as intelligence is a measure of “an agent’s ability to achieve goals in a wide range of environments”.^21^ This definition encompasses a wide variety of systems from artificial to biological, and if we take “goal” or intent to be the maximizing of Darwinian fitness this means intelligence is a fundamental property of life.^32,33^ This definition also implies the ability of problem-solving as a core emergent property of intelligence. Given this definition and the amassed evidence, the question is not whether plants express intelligent behavior but how they achieve it without a nervous system and what the ecological consequences of these behaviors entail.

**Figure:**
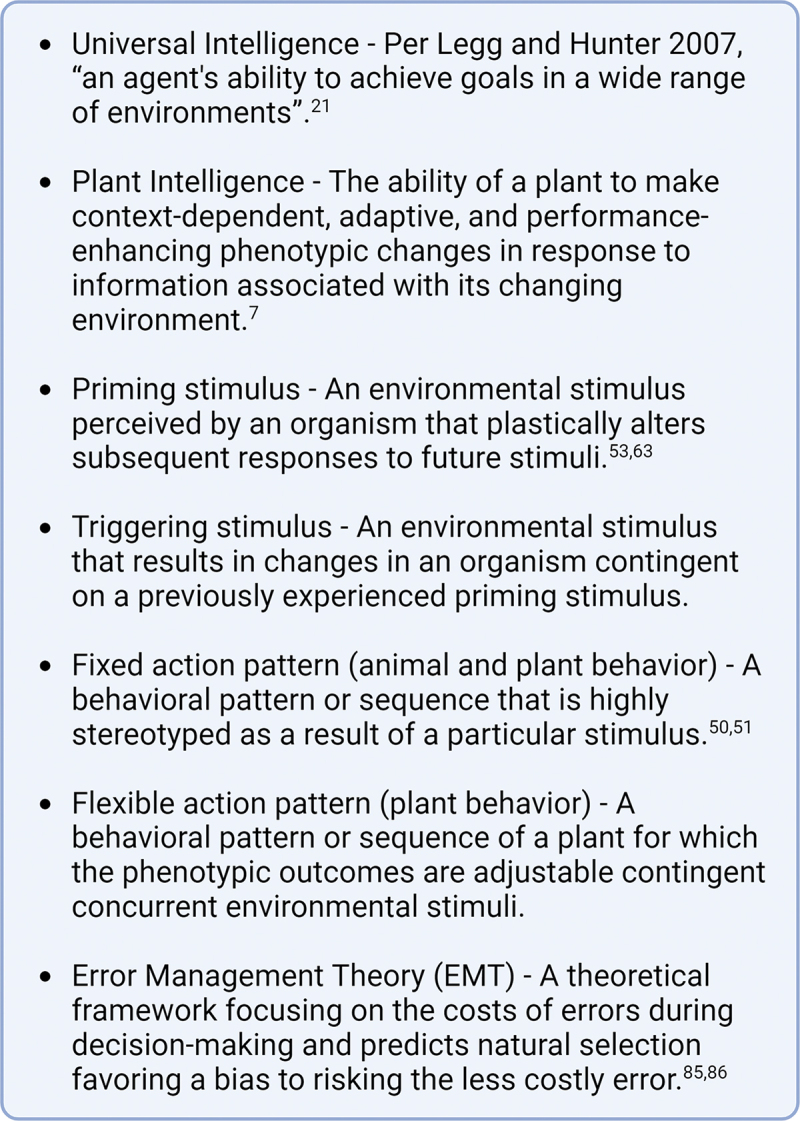

Specifically applied to plant behavior in the context of plant–herbivore interactions, intelligence would be apparent in the context-dependent, adaptive, and performance-enhancing phenotypic plasticity a plant expresses in response to information associated with its changing environment (Box 1). This information can be in the form of previous experiences (e.g. plant immunological memory, internal and external priming), acute alterations of environmental factors, or anticipated changes in the environment (plant–plant communication about herbivory and competition). For induced responses to herbivory, the respective information exchange is, to a significant proportion, mediated by chemical signals both to systemically alter plant metabolism (endogenous signaling) and affect mutualistic and antagonistic interactions with other organisms. Moreover, the environmentally induced alteration of secondary metabolite production expands the interaction arena of a plant and provides some of the most striking examples of plant behavior.^3,34^ The interest in alternative conceptual frameworks to study plant phenotypic plasticity is not solely due to the refinement of instruments to measure ‘other’ or slower behavior but also because the consequences of the behavior of primary producers can disproportionately impact community and ecosystem dynamics.^3,35^ While no direct evidence for that is available yet, a plant’s ability to adjust its phenotypes to interacting environmental factors has been suggested as a major factor influencing globally important ecosystem and biogeochemical dynamics.^24,25,31^

Because plants perceive their environment, and in particular their biotic environment, to a large extent using chemical cues, understanding plant behavior requires an understanding of the information encoded and transferred with chemistry. Among the most obvious information-carrying plant secondary metabolites, are volatile organic compounds (VOCs) constitutively and inducibly emitted by plants. Volatile organic compounds have been found to mediate many types of ecological interactions from mutualistic interactions with pollinators and natural enemies of herbivores (indirect defenses) to directly defending against antagonists, such as herbivores and competitors.^9–11,36^ Here we use chemically-mediated information transfer in general, and that mediated by VOCs in particular, within a plant–herbivore interaction context as a model for how environmental information is integrated to mediate “intelligent” behavior and how such behavior is affecting higher-level ecological dynamics. Based on the above-mentioned general definition of intelligence, that of “an agent’s ability to achieve goals in a wide range of environments” (Box 1), we focus our analysis on studies that show a plant’s ability to express alterations in response to insect herbivores and how these responses can be influenced by a plant’s past and present experiences but also shaped by the plant’s relative perception of future risk. We also seek to address the quagmire of dialogue on the concept of plant intelligence, namely is the framework not only appropriate but also useful?^7,17,29,30^ The ultimate short-term scientific value of a concept would seem to be the framework’s ability to generate new testable hypotheses, and the long-term scientific value of the outcomes and relevance of these inquiries.

## Context dependency of plant endogenous signaling pathway crosstalk

Herbivore attack induces complex, yet predictable, changes to plant primary and secondary metabolism. These responses are mediated by interactions between herbivore-derived elicitor compounds and plant endogenous and phytohormonal signals.^8^ These changes can be highly specific to the attacking herbivore and further influenced by other biotic and abiotic factors.^37,38^ Both, the high specificity and the alteration by environmental factors are thought to result from phytohormonal signaling pathway crosstalk allowing for the fine-tuning of transcriptional and metabolic reconfiguration of the plant when under attack.^8^ While this endogenous signaling pathway crosstalk involves a large diversity of phytohormones and phytohormone-like compounds, research has established the clearest evidence for the interactions between three crucial endogenous signaling pathways associated with inducible resistance to herbivores and pathogens: jasmonates (JA), salicylates (SA), and ethylene.^8,39^

Most relevant to this review is that these phytohormones can function synergistically or antagonistically when regulating a plant's metabolic response, depending on the context, that is, the attacking herbivore or pathogen.^40^ Moreover, whether or not a phytohormonal signaling interaction can be categorized as synergistic or antagonistic depends on the downstream metabolic change (e.g. in secondary metabolism) examined, and consequently the herbivore/pathogen affected by that change. For example, like in many other plant species, in the wild tobacco, *Nicotiana attenuata*, induced accumulation of JA and ethylene signals in response to the attacks by pathogenic fungi synergistically interact to increase the accumulation of fungicidal phytoalexins.^41^ A similar synergistic interaction between JA, ethylene, and herbivore-derived elicitor compounds has been found to regulate the induction of VOCs from maize plants that are attacked by *Spodoptera exigua* caterpillars.^42^ In contrast, when caterpillars of the specialist tobacco hornworm, *Manduca sexta*, attacks *N. attenuata* plants, the simultaneous activation of JA and ethylene signaling pathways results in the attenuation of insecticidal nicotine production, indicating an antagonistic interaction of jasmonate and ethyl signaling.^43^ These seemingly opposing outcomes of the crosstalk between the JA and ethylene endogenous signaling suggest that the interaction between two different signaling pathways can be realized at different points in the induction process (from the perception of an environmental cue to the expression of resistance mediating metabolism) and that this can be mediated by differential activation of phytohormone-regulated promoters. It also serves to highlight the point that crosstalk between phytohormonal signaling pathways and their differential outcomes can be evolutionarily adapted and behaviorally adjusted to fine-tune plant metabolism to meet the challenges of the current environmental contexts.^44^

Like the Ethylene-JA signaling pathway crosstalk, the interaction between JA and SA-mediated signaling is deeply intertwined with land plant evolution but has been found to be predominantly antagonistic.^45^ The dominant paradigm states that while SA signaling regulates systemic acquired resistance to biotrophic pathogens, viruses, and some species of sap-feeding insects, JA signaling largely mediates induced resistance to chewing (tissue-damaging) herbivores, necrotrophic fungi, bacteria, and nematodes.^40,46^ Moreover, this phytohormonal antagonism largely results from negative transcriptional regulation of one phytohormone on the other’s pathway and associated metabolic effect.^40^ Although specific pathogens or herbivores individually can trigger both signaling pathways, JA and SA regulate distinct metabolic responses that can differentially affect plant antagonists.^44^ However, already early on in the study of the JA-SA crosstalk, researchers found that, as with the Ethylene-JA interaction, the expression of antagonism vs. synergism is a function of the specific metabolic changes regulated, the sequence in which the signaling pathways are activated and the strength of induction. Consequently, the pathway crosstalk can be hypothesized to optimize the defense against a specific attacker. For example, experiments with domesticated tobacco (*Nicotiana tabacum*) and Arabidopsis (*Arabidopsis thaliana*) interacting with a common bacterial pathogen (*Pseudomonas syringae*) suggested that there was a transient synergistic interaction between SA and JA signaling at low phytohormonal concentrations that led to the increased expression of defensive genes regulated by both pathways. At higher concentrations, however, both pathways triggered fundamentally different, deleterious plant responses, including the accumulation of reactive oxygen species and death.^47^ Similarly, functionally synergistic effects between SA and JA pathways seem to be at play in plant interactions with biotrophic pathogens. Plants can very efficiently overcome attacks by biotrophic pathogens by initiating SA signaling-associated programmed cell death near the initial infection site because this limits the spread of the pathogen.^48^ However, the now-dead cell material can become more susceptible to necrotrophic pathogens. In contradiction to the commonly found JA-SA signaling antagonism, a study with *Arabidopsis* found that the biotrophic pathogen-induced SA accumulation increased the expression of JA-responsive genes as well as *de novo* JA accumulation and so mediates systemic acquired resistance in *quasi* anticipation of an inevitable invasion by a necrotrophic pathogen. This synergistic interaction of SA and JA signaling was interpreted as a mechanism through which plants can be defended against biotrophs while not becoming more vulnerable to necrotrophs.^49^ Again, the response to one specific attacker was fine-tuned, however, this time seemingly not only to that initial attacker but also to one that is most likely to follow.

## Intelligent plant responses to herbivores: fixed action patterns or flexible adjustment of defense phenotypes

Does a conceptual framework of intelligent plant behavior affect how we would view and thus further study these kinds of specific plant responses to their attackers? According to the framework, the first question we would have to ask is the one for the ultimate goal and some “purpose” of the specific behavior. Universally, the ultimate “goal” for any organism as demanded by the evolutionary mechanism of natural selection is maximizing fitness. Thus, a particular plant behavior, or more specifically, an optimized response to a stressor in the context of additional environmental cues and stimuli, has to be evaluated for its eventual contribution to maximize the fitness outcome under different environmental circumstances. This means that signaling pathway crosstalks can be predicted to have different outcomes for a specific plant–herbivore interaction when the environmental context demands.

On one hand, these differential outcomes can be a result of natural selection on specific pathway crosstalk patterns adapted to a predictable frequency and composition of environmental conditions, an innate behavior that is genetically determined rather than affected by (learned through) the experience of an individual. In animal behavioral biology, such relatively invariant, stereotype behaviors are termed fixed action patterns (Box 1).^50,51^ In support of such a fixed action pattern hypothesis, recent studies on the Brassicaceae annual *Brassica nigra* found that plants are predispositioned to express induced responses to common patterns of sequential herbivore attackers and the most prevalent herbivores rather than specifically to a first attacker.^44^ This remarkable finding could explain why many plant responses to specific herbivore species do not follow the predicted JA and SA-mediated induction patterns that are commonly found in standard interaction bioassays and which usually do not consider the sequence or relative importance of interacting herbivore species.^52^ Moreover, these studies suggest local adaptation of the induction patterns to specific community contexts.

**Figure 1.**
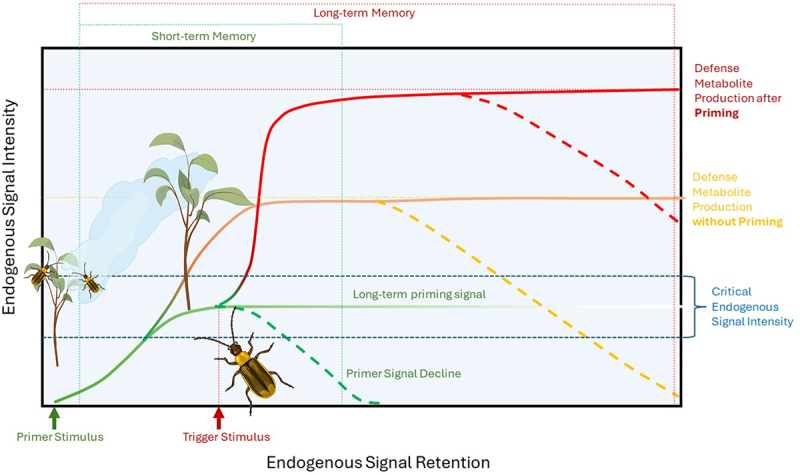
Plant defense priming in response to herbivory. Primer stimuli are environmental cues (e.g. volatile organic compounds from damaged neighboring plants, direct herbivore damage, spectral and chemical information) that elicit plant endogenous signaling and so ready plants for faster and stronger responses when additional attacks by herbivores occur (trigger stimulus). Intensity of the priming stimulus and the plant’s inherit sensitivity determine how strongly the plant is responding to a stimulus, reaching from alterations in endogenous signaling that may not significantly affect metabolism to a direct induction of defense metabolism. If the endogenous signal intensity elicit by environmental stimuli ranges within a critical signal intensity, a subsequent trigger stimulus (e.g. direct damage by a herbivore) will result in a faster and stronger expression of the plant defense metabolism. The reliability of a priming stimulus as a predictor of subsequent fitness-affecting damage will affect endogenous signal intensity and retention and thus if the priming information is stored in short- (e.g. transient, transcript and phytohormone accumulation) or long-term memory (e.g. epigenetic alterations). Defense priming allows the integration of environmental information to optimize plant responses while minimizing the costs associated with unreliable (false) environmental information.

As an alternative to such a fixed action pattern that is shaped by predictable interaction community dynamics, varying environmental conditions could directly affect the expression of signaling pathways or the genes regulated by them and so lead to a differential outcome of the signaling pathway crosstalk in different environmental contexts (e.g. flexible behavior). In support of this hypothesis, plants can be expected to alter their induction patterns based on interacting cues in the environment in a far more flexible way, based on experience and the interpretation of cues predictive of future conditions. So-called priming effects would fall into this category as they allow plants to utilize environmental cues to ready behavioral adjustments without a costly investment into a full-out metabolic reconfiguration (Figure 1).^53^ These could be A) previous direct inductions of transcriptional or metabolic changes that alter the responses to a subsequent attacker or B) alteration of responses after the plant has perceived non-damaging environmental cues such as spectral and chemical cues from interacting organisms, such as neighboring plants or attackers that may predict future competition or herbivory, respectively. We will discuss such priming effects as immunological memory and predictive environmental information, respectively, below (Figure 1). For now, let’s stay with the major prediction that we can derive from the two hypothesized types of intelligent plant responses to herbivory.

Both, induced responses based on locally adapted fixed action patterns as well as those that are based on more flexible adjustments of standard responses, predict environmental context-dependent alterations of signaling pathway crosstalk of the kind that we have highlighted above. The difference would lie in the way phytohormonal expression, and the defense gene transcription is regulated. More importantly, however, the fitness outcome of interaction and the associated metabolic reconfiguration of the plant can be much more dependent on the environmental context. It would thus challenge the paradigm of inducible plant defenses functioning as a cost-saving strategy.

Metabolic changes in response to herbivory are frequently found to cause increased resistance and so affect the currently attacking as well as subsequently approaching herbivores and reducing future risk of herbivory.^8^ There are three major ways through which induced responses in secondary metabolism mediate resistance.^3,34^ A) The induced production of toxic, antidigestive, antinutritive, and repellant compounds can reduce the attractiveness and palatability of the plant tissue and thus compromise the presence and performance of herbivores on the plant (induced defenses as a cost-saving strategy). B) Altered compositions and diversity of compounds can confuse herbivore host searching behavior or affect the functionality of secondary metabolite mixtures (moving target hypothesis).^54^ C) Compounds, such as VOCs induced after herbivory can function as information to herbivores to indicate increased resistance status of the plant or to natural enemies of herbivores (predators and parasitoids) and facilitate their host/prey search behavior and so indirectly affect plant performances positively through the indirect reduction of herbivory (chemical information hypothesis).^3,55^ Within the cost-saving paradigm, inducible direct resistance allows plants to escape the potentially high costs of consistently expressing defense-mediating traits. These costs can take the form of either direct metabolic resource allocation costs or ecological costs that arise from defensive chemistry compromising interactions with mutualists, such as natural enemies and pollinators.

This cost-saving hypothesis of inducible defenses commonly refers to the costs saved relative to the constitutive expression of the respective defense trait. Indeed, numerous studies demonstrate that the constitutive expression of an otherwise inducible trait can be very high^56,57^ and the apparent trade-off between constitutive and inducible defenses is one of the most commonly found patterns in the study of plant–animal interactions.^58,59^ However, both the metabolic costs, as well as the ecological costs, that could be associated with inducibility *per se* can be significant and may match or even exceed the costs associated with the constitutive expression of defense traits. For example, a study silencing wound signaling-associated mitogen-activated protein kinases and thus inducible resistance in wild tobacco, *Nicotiana attenuata*, revealed significant allocation costs of maintaining the wound signaling mechanisms.^60^ Similarly, the inducibility of herbivore resistance and the associated VOC-signaling led to herbivory-induced pollinator limitation in the wild tomato *Solanum peruvianum* and was initially interpreted as high ecological costs of inducibility.^61^ The apparent ecological cost of inducible resistance compromising interactions with pollinators is thought to drive even macroevolutionary patterns. A comparative study of the Solanaceae plant family found self-compatible species (e.g. less reliant on pollinators) generally expressing stronger inducible defenses while self-incompatible species relied on constitutively high (less inducible) defenses.^59,62^ However, why then does the self-incompatible wild tomato, *S. peruvianum*, express high inducible defenses when, as a result negative consequences on pollination could be expected? The herbivory-induced pollinator limitation observed in this species turns out to stabilize the population dynamics of all involved interactors, so minimizing the occurrence of severe fitness losses.^12^ Thus, the plant’s behavior maximizes long-term gains for potential short-term losses.

In conclusion, the potentially high costs of inducibility as well as the integrative function of inducibility in manipulating the plant’s interaction community suggest induced resistance less as a cost-saving strategy but rather as an alternative strategy to a constitutive expression of respective traits, whereby the viability of either strategy is determined by life history demands as well as the environmental context. Inducibility provides metabolic flexibility that can optimize metabolism to maximize fitness and survival in variable environments. In addition, inducibility provides specific information about a plant’s metabolic status and so can affect interactions with antagonists (e.g. chemical aposematism) and mutualists (e.g. indirect defenses), expanding the arena of plant interactions to the benefit of the plant.^3^

## Plant memory mitigates the potential costs of inducing responses based on false information

Numerous studies have reported that previous exposure to a variety of stressors or cues alters plants’ response to subsequent herbivory.^53,63^ Such dual-stage responses are commonly referred to as priming and have received attention for their potential applications in pest control and their implications on plant signaling and behavior (Figure 1).^64,65^ The initial primer stimuli fall along a continuum of intensity and resulting effects, roughly reaching from direct tissue damage or metabolic stress to the perception of environmental cues that do not directly trigger a stress response, such as the perception of chemical or physical cues from other interacting or neighboring organisms, such as neighboring plants, nonpathogenic rhizobacteria, symbiotic fungi, or not directly interacting arthropods.^66–73^

It is commonly found that an initial experience of stress by the plant (primer stimulus) can lead to significant differences in how plants respond to subsequent trigger stimuli, including in A) the quantitative expression of defense traits, B) the speed with which induction maxima are reached, and C) the specificity (quality) of the response when a plant receives a second stimulus, such as an attacking herbivore (Box 1, Figure 1).^53,65^ Accordingly, the mechanisms through which priming alters subsequent responses seem various but dependent on plants being able to memorize the primer stimulus. How plants store information about previous attacks has become one of the most active fields in the research of plant-induced responses to biotic and abiotic stressors. Recent studies found evidence for plant immunological memory being mediated by A) epigenetic changes, such as DNA methylation, histone modification, small RNA-mediated gene silencing, and chromatin modifications, B) transient defense gene transcript accumulation, and C) endogenous defense signaling compound accumulation.^69,74–81^ Thereby, epigenetic changes can persist for longer periods and can, through maternal effects, mediate even transgenerational defense priming. For example, in *Arabidopsis thaliana* plants, small interfering RNAs mediate the priming of JA-dependent defense responses for up to two generations as the epigenetic alterations are transferred from the mother plant to the seeds.^77^ Thus, such epigenetic priming mechanisms can be understood as mediating long-term memory that persists through newly developing tissues and into subsequent generations. In contrast, priming mediated through transient transcript or phytohormone accumulation can, by their nature of activity, only function transiently as short-term memory. Thereby, multiple memory mechanisms are likely simultaneously at play in an individual plant, mediating long and short-term memory and differentially regulated metabolic and growth responses when a trigger stimulus is applied (Figure 1).

**Figure 2.**
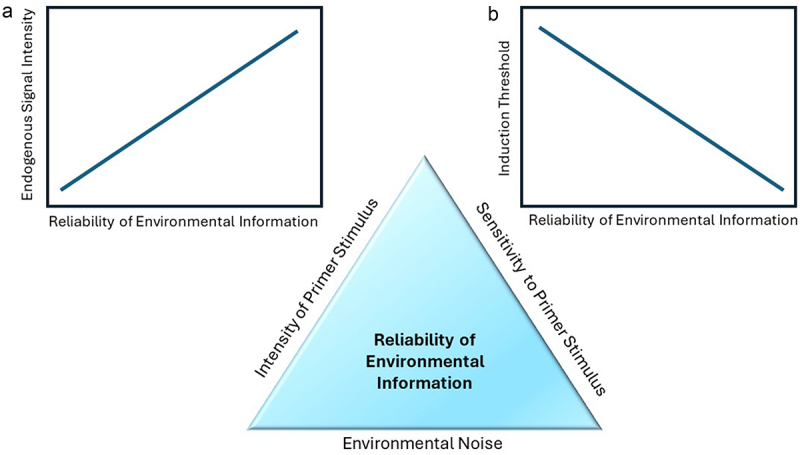
The predictive value of environmental information to the plant defending against herbivory. The reliability of environmental information lies in the intensity of the primer stimulus, the plant’s sensitivity to the primer stimulus, and the environmental noise obstructing information transfer. Plants should evolve stronger priming responses (endogenous signaling) to more reliable environmental cues (A), while reducing the threshold for direct induction of defense-related metabolic changes (B). The threshold is determined by the zone of critical endogenous signal intensity, within which a trigger stimulus will induce stronger and faster responses and above which resistance is directly induced.

How important is the ability for plants to memorize previous environmental stresses and so be able to prime for subsequent attacks? On one hand, it seems intuitive that if plants can respond stronger or faster to a new stressor when previously having been attacked could convey some advantage, as the increased resistance presumably reduces the negative impact of the subsequent attacker. On the other hand, however, the initial induction of responses by the primer stimulus can be associated with costs that come from a herbivore’s consumption of photosynthetically active tissues and the induced metabolic reconfiguration of the plant. The costs of plant defense priming are considered relatively low because primer stimuli are assumed to be associated with a minimum of tissue damage, but they are also very rarely measured.^82^ In one such rare case, the chemical priming of *Arabidopsis* plants with low amounts of the resistance elicitor beta-aminobutyric acid (BABA) against pathogen attacks reduced plant growth rates but did not affect final seed production, while making the plant more resistant to subsequent pathogen attacks than untreated plants. However, higher amounts of BABA, directly induced resistance, but had significant negative effects on both growth and seed set.^83^ Thus similar to the costs of induced resistance, the net costs of priming are a function of the magnitude of the initial induction and the benefit gained from reducing future damage.^84^ These benefits of priming, in turn, depend on the advantages gained by an accelerated or stronger future response, as well as the probability and risk of damage severity of encountering future herbivory and so the predictive nature of the priming stimulus (Figure 2). This also means that the evolution of priming of plant defense responses, like inducibility in general, can be hypothesized to be driven by the costs of acting on false information.

Signal detection theory provides a framework to address this problem of decision-making under uncertainty and differentiates four possible types of information on which an individual’s decision outcome is measured: true positive, true negative, false positive, and false negative.^85^ This concept implies the evolution of strategies that allow mitigating errors resulting from inducing responses based on false information (error management theory (EMT)). EMT has been applied to understand under what conditions plants should evolve induced resistance vs. expressing constitutive resistance (Box 1). EMT focuses on the costs of errors during decision-making and predicts natural selection favoring a bias to risking the less costly error.^86^ Accordingly, plants that can suffer from significant costs of herbivory in just a single event (e.g. plants in the seedling stage, plants exposed to regularly outbreaking herbivore populations, plants in environments with consistently high herbivory) should err on the side of producing strong defenses even if they might not be needed, such as unnecessary high constitutive defenses (bias toward false positive errors). As the costs associated with being consumed by a herbivore decline, plants can afford to err on the side of acting on false negatives, meaning to rely more on inducible resistance, even if that means the plants might not be appropriately defended against an attacker most of the time.

Priming, and so readying defenses in anticipation of a second attack, can behaviorally mitigate the potential costs associated with false information. This is because the quality and magnitude of the priming stimulus can predict the probability of future attack and thus the relative cost of error associated with either direct or delayed induction to a trigger stimulus.^53,65^ This behavioral adjustment to the predictability of future damage can allow plants to take advantage of readying defenses without a significant investment of resources into constitutive defenses or even risk the resource allocation and ecological costs associated with an immediate induction of resistance.^69^ This applies to priming by mild damage and would be even more pronounced when plants ready their defenses after perceiving non-damaging cues that are predictive of future damage, such as VOC from damaged neighboring plants.^87^ Moreover, the net costs of herbivory usually interact with impacts from other antagonistic interactors, most notably competitors and pathogens.^88,89^ Priming allows plants to fine-tune responses by integrating current conditions and acting on past information that is predictive of future conditions without a significant up-front cost of utilizing this information (Figure 2). Moreover, priming mediates the adjustment of standard behaviors to fluctuating environmental conditions toward the goal of maximizing reproductive output, fulfilling the most general definition of intelligent behavior as applied to plant behavior. A recent modeling approach to integrating EMT into plant defense theory and priming, specifically revealed that optimal priming is a function of the level of competition, herbivore feeding rate (risk of severe damage), the time between priming and trigger stimulus, and plant life history traits (e.g. time for which the plant stays primed, plant age, and the response pattern of the primed plant).^90^ The study revealed that in order to assess if plants indeed optimize priming responses, we need to know more about herbivore dynamics and how they affect plants as well as the predictive value of priming stimuli. Such data are very limited at present but recent studies into how plants use non-damaging environmental cues to predict future events provide an emerging framework of hypotheses on how plants integrate information to optimize behavioral responses to antagonists.

## Integrating environmental information to anticipate future conditions

Priming stimuli that alter a plant’s future responses to antagonists without inflicting any tissue damage are special as they can be based on partially abstracted information that needs to be predictive of future conditions to affect plant performance. Although it seems unlikely that plants are planning for the future in a similar way to some animals, they do in fact make anticipatory changes to their morphology, chemistry, and transcriptome in response to environmental cues.^6,91^ As defined by Mertens et al. (2021) anticipatory changes in plants are a “response to information indicative of stress in which the phenotype is adjusted in anticipation of suboptimal conditions or arrival of stress”.^52^ These anticipatory changes seem to be ubiquitous in plants and likely arise from the nonrandom nature of interactions as influenced by plant or herbivore phenological and ontogenetic change, optimality of abiotic conditions, or the probability of simultaneous or sequential attack.^6,44,52,92–94^ The direct costs associated with metabolic responses to those stimuli are assumed to be minimal, leaving the potential costs as well as the relative benefits determined entirely by the predictive value of the information and the errors associated with acting on false positives and negatives. A major prediction of EMT is that the direct metabolic response to such a priming cue should scale with its predictive value (Figure 2). Studies on plant-plant communication can give a remarkable first insight into this issue. For example, wild tobacco plants, *Nicotiana attenuata*, are more resistant to herbivory when exposed to VOCs from clipped (mechanically damaged) neighboring sagebrush plants, *Artemisia tridentata*. ^69,95^ This was reported as one of the first cases of eavesdropping on a neighboring plant’s wound signaling in nature and provided clear evidence of increased resistance in the tobacco plants responding to chemical information from neighbors.^95^ However, in most of the distribution range, these two plant species do not share the same herbivores, and those that are shared, inflict minimal damage.^69^ Thus for tobacco plants, the chemical information coming from damaged sagebrush plants has little predictive value, risking high costs if resistance is directly induced in response to the priming cue (false positive). In such cases, EMT would predict no priming effect at all or no direct induction of metabolic changes in response to the priming cue. Indeed, a metabolome analysis revealed no direct induction of any defense-mediating secondary metabolites in sagebrush VOC-exposed tobacco plants. Yet, increased accumulation of defense gene transcripts in response to damaged sagebrush-VOC exposure primed tobacco plants for a faster and stronger expression of anti-digestive proteinase inhibitors upon attack (trigger stimulus) and explained the increased resistance of exposed plants.^69^ Thus tobacco plants utilize short-term priming mechanisms to minimize the probability of false positives while minimizing the costs associated with and opportunistically taking advantage of information about potential future attacks. As the predictive value of this information increases, the priming stimulus should result in more direct induction of metabolic changes. Unfortunately, information about how either *N. attenuata* or *A. tridentata* plants respond to their own presumably more predictive damage-induced VOC emissions (but see^96,97^ and the associated levels of priming are limited and not conclusive. However, recent findings from tall goldenrod, *Solidago altissima*, indicate a more direct induction of chemical defenses when information is more predictive.^98^ Different from the previously described species, *S. altissima* occurs in very dense, well-connected populations. Strong inducible resistance in combination with VOC-mediated plant–plant information transfer about herbivory allows plants in these populations to share the risk of herbivory with their neighbors, thus minimizing the damage that each individual plant in the population receives. ^98–100^ In consequence, herbivory selects for a more open information transfer between plants of the population. Most importantly, VOCs from damaged *S. altissima* plants directly induce the production of at least part of the defensive secondary metabolism suggesting that in systems where the predictive value of the priming stimulus is high, the priming stimulus can directly increase defense metabolite production as well as accelerate and amplify the response to a subsequent trigger stimulus (actual herbivory).

Here it is important to consider that VOC-mediated information transfer not only entails potential costs of acting on false information for the receiver. The evolution of VOC-mediated plant–plant interaction is commonly thought to be primarily driven by the costs paid by the emitter for providing the information. Eavesdropping on a neighbor’s herbivore-induced VOC emission can provide the receiver with a competitive advantage over the emitter. Depending on the relative costs for the emitter of information, natural selection may favor the production of VOC blends that obscure the information content for the receiver, consequently resulting in the evolution of private channel communication. The term private channel communication is used when information is restricted to selecting interactors. In contrast, open channel communication is evident when information can be processed and understood by a wide variety of receivers, such as the information that is transmitted with general alarm cues.^87^ Studies in *A. tridentata* demonstrated a high chemotype and kin specificity of VOC-mediated resistance induction suggesting the existence of such private channel communication that favors information transfer within similar chemotypes and close relatives but minimizes transfer between distantly related plants.^97^ In contrast, in *S. altissima*, where inducibility and information transfer interact to minimize herbivory, emitters converge on a more similar VOC bouquet upon damage allowing the transmission of information to be open to all interactors in the population. However, when *S. altissima* populations are released from herbivory and plant–plant communication becomes less beneficial natural selection favors private channel communication again.^100^

In addition to a stronger response to priming, the hypothesis of flexible intelligent plant behavior and EMT predicts higher specificity of the transferred signal and the elicited responses as the error associated with priming decreases. Moreover, such increased specificity can be expected to be evident in how plants respond to a specific herbivore as well as in how cues about the environmental context (e.g. other herbivores, competitors, abiotic conditions, and natural enemies of herbivores) affect the perception and processing of the priming stimulus. Specificity to an attacking herbivore has recently been demonstrated in a Californian coastal shrub, *Baccharis salicifolia* (Asteraceae). VOCs from aphid-damaged plants would induce resistance in a neighbor only to the same aphid species that had elicited the VOC emission.^101^ However, more comparative studies are needed to assess natural variation in the specificity of priming as well as its fitness effects on the plant.

Environmental context specificity is evident in cases where other environmental cues affect the perception and processing of a priming cue. A pertinent example of this is the plants’ use of spectral biotic information about neighboring plants to make anticipatory defensive changes.^102^ For example, plants can anticipate future competition based on the light quality reflected off of neighboring plants. As full-spectrum light is absorbed by plants, chloroplasts preferentially absorb red light. This preferential absorption causes a shift toward far-red in the light reflected off or transmitted through leaf surfaces.^103^ By monitoring shifts in the ratio of red to far-red light plants can perceive and anticipatorily respond to not-yet-experienced competition by making several morphological and chemical changes.^104^ One of the more subtle changes plants seem to make in response to anticipated competition is the attenuation of constitutive and induced defenses.^102,105^ Interestingly, recent studies with domesticated tomato, *Solanum lycpersicon*, demonstrated that decreased red:far red light ratio perception does not only attenuate direct constitutive and inducible resistance but causes an increase in herbivory-induced emission of VOCs. Because these increased VOC emissions attract predatory insects to the plant, which can reduce herbivory, this was interpreted as a shift of resources not only from direct defense to competition but also from direct defense to indirect defense. The first shift represents a plant’s behavioral avoidance of the error of not responding to a potentially competitive neighbor, thought to be more costly than the expression of direct defenses (acting on the less costly false negative). Similarly, the second avoids relatively costly direct defenses in favor of less costly indirect defenses. These trade-offs were originally thought to be mediated by direct plant internal resources competition, but this recent work has shown that these responses can become experimentally uncoupled, suggesting that they are more influenced by “regulatory decisions” that allow plants to fine-tune their phenotypes relative to experienced environmental conditions.^84,106–109^ In light of such priming of responses through the integration of diverse environmental cues, it is interesting to consider potential alternative functions of the altered VOC emission when *S. lycopersicn* plants integrated spectral information about their neighbors with the chemical information from herbivory. For example, in some plant species, such as the above-mentioned *S. altissima*, VOC-mediated priming of direct defenses seems a much more fitness-impacting function than indirect defenses raising the question if VOC-mediated information of oncoming herbivory is similarly integrated with spectral information as direct herbivore damage to the plant tissues. Indeed, a recent study on *S. altissima* found that herbivory-induced VOC emission is significantly altered when plants are exposed to spectral cues from neighbors. More importantly, plants that are exposed to spectral cues from neighboring plants seem to perceive VOCs differently when inducing direct defense metabolite production, suggesting integration of information on both the emitter as well as the receiver side.^50^ The seemingly adaptive behavioral adjustment of priming and induction processes in the light of such fundamental trade-offs between the investment into different defense strategies and competition meet the basic definition of intelligence in that it overrides the full induction of defense when it could be potentially maladaptive in a competition scenario. It also reiterates that a plant that can integrate different types of environmental cues in anticipation of future conditions during the priming process, can minimize errors in defense induction altogether.

## Conclusion

As our outline above suggests, several already-known phenomena associated with plant-induced responses to herbivory can be understood as and analyzed under the umbrella of a general concept of intelligent behavior. However, this framework must be evaluated by its ability to generate new hypotheses and predictions. The predictions that we can extract from our review are largely about the mechanisms that allow the integration and processing of environmental information and ecological functions of chemical information transfer. On the functional level, we hypothesize priming of defense responses that integrates multiple types of environmental cues as a way for plants to optimize defense expression in accordance with their developmental status and environmental circumstances. Thereby the ability to prime for, rather than directly induce resistance, allows plants to minimize the potential costs associated with false responses to environmental information. This, for example, predicts that plants under different environmental conditions (e.g. competition vs. no competition) should prime and induce resistance differently to the same herbivore species, depending on the relative costs associated with the different types of interactions (here, herbivory vs competition). It also implies that plants could evolve fixed action defense patterns if the environment is relatively predictable, or behavioral flexibility with highly specific inducibility when plants experience variable environmental conditions. For example, such increased flexibility in the priming and induction of responses can be predicted for species that have wide distribution ranges across broad latitudinal and elevational gradients in intermixing meta-populations. In contrast, pronounced local adaptation patterns of specificity of induced responses to herbivory can be predicted if plant responses are based on fixed action patterns. On a mechanistic level, we hypothesize that the differential expression of plant intelligence is based on the plant’s ability to store and process information that is predictive of future risk of attack. This framework differentiates between long-term and short-term memory in plants that are mediated through different molecular mechanisms (epigenetic vs. transcript and phytohormone accumulation, respectively) yet interact to regulate priming and eventual induced resistance responses. Finally, the outcome of signaling pathway crosstalk can be expected to be far more flexible than the current paradigm suggests and should vary with the environmental information processed, but also by which promoters are regulated by endogenous signaling pathways and at what junction in the perception to transcriptional and metabolic reconfiguration process of the plant.

It should be clear that even if we consider plants that respond to herbivory with context-dependent, adaptive, and performance-enhancing phenotypic changes as intelligent, this still might constitute a different type of intelligence than that attributed to single-celled organisms, fungi, and animals (including humans). However, all life can be considered to have the same goal of successfully reproducing, making the problem-solving capacity of intelligence converge on a similar purpose for all life. This is obviously in contrast to artificial intelligence which has not yet been given or seemingly acquired the drive of self-proliferation, and with its goals being defined by ephemeral human desires.
